# Clinical Utility of Circulating Tumor Cells in Patients With Esophageal Cancer

**DOI:** 10.3389/fonc.2022.828368

**Published:** 2022-03-21

**Authors:** Yang Li, Zhenxing Wang, Rao Fu, Shuang Wang, Tingting Zhang, Xudong Tian, Dawei Yang

**Affiliations:** ^1^ Zhong Yuan Academy of Biological Medicine, Liaocheng People’s Hospital, Liaocheng, China; ^2^ Department of Thoracic Surgery, Liaocheng People’s Hospital, Liaocheng, China

**Keywords:** esophageal cancer (EC), circulating tumor cells (CTCs), NE-FISH, diagnostic, prognostic

## Abstract

**Background:**

As one of the most aggressive gastrointestinal tract cancers, esophageal carcinoma (EC) had the tenth morbidity and sixth mortality rate globally in 2020. This study was conducted to investigate whether circulating tumor cells (CTCs) could be used as diagnostic and prognostic tools for patients with EC.

**Methods:**

Peripheral blood samples were collected from 129 patients newly diagnosed with EC, 17 individuals with benign diseases, and 75 healthy donors for CTC analysis using the negative enrichment-fluorescence *in situ* hybridization (NE-FISH) approach. The relationship between CTCs (counts and karyotypes) and clinicopathological features was then investigated. Moreover, overall survival (OS) and progression-free survival (PFS) were analyzed to evaluate the predictive value of CTCs.

**Results:**

The detection of CTCs using the NE-FISH approach helped in differentiating patients with EC from benign or healthy controls at a threshold of 2 per 3.2 ml peripheral blood with a sensitivity and specificity of 70.54% and 96.74%, respectively (area under the curve = 0.826, 95% CI 0.770–0.874, *p* < 0.001). The CTC count was associated with tumor depth (*p* = 0.012), but there was no correlation with other clinicopathological characteristics. Furthermore, the proportion of CTCs with chromosome 7 triploidy was linked to distant metastasis (*p* = 0.033) and TNM stage (*p* = 0.002). The OS was significantly shorter for patients with CTCs ≥ 3 than for those with CTCs < 3. Univariate analysis revealed that sex, vascular invasion, distant metastasis, tumor depth, lymph node metastasis, and TNM stage were the significant prognostic factors for patients with EC. Multivariate analysis demonstrated that distant metastasis (hazard ratio (HR) 3.262, 95% CI 1.671–6.369, *p* = 0.001 for PFS; HR 3.759, 95% CI 1.867–7.571, *p* < 0.001 for OS) was a significant prognostic factor for patients with EC.

**Conclusions:**

Detection of CTCs using NE-FISH could be helpful in the diagnosis of EC. The proportion of CTCs with chromosome 7 triploidy was related to distant metastasis and TNM stage. Patients with CTCs ≥ 3 had short OS, while distant metastasis was an independent factor indicating a poor prognosis for patients with EC.

## Introduction

Esophageal cancer (EC), a common gastrointestinal cancer originating from the esophageal epithelium, was the tenth most prevalent tumor, with 604,100 new cases, and the sixth primary cause of tumor-related deaths, with 544,076 deaths, worldwide in 2020 (1). Endoscopic screening, imaging examination, and detection of serum protein tumor markers are helpful in the diagnosis of EC. However, early diagnosis remains to be tricky because the symptoms are less specific (2, 3). Owing to the properties of insidious onset, highly invasive nature, and rapid progress, the disease is already at an advanced stage, with regional or distant metastases, in a considerable proportion of the diagnosed cases. Thus, the opportunity for surgical resection is missed. An effective adjuvant diagnostic method is hence required to detect EC at an early stage, especially in asymptomatic patients.

As one of the most promising and wildly used biomarkers in liquid biopsy, circulating tumor cells (CTCs) are tumor cells that escape from primary or metastatic lesions into the circulatory system after epithelial–mesenchymal transition (EMT) and result in tumor metastasis or recurrence (4, 5). CTCs detected *via* a non-invasive method can be used as a biomarker for cancer diagnosis, metastasis prediction, recurrence monitoring, and therapeutic response assessment in various malignancies such as gastric, breast, colorectal, and lung cancer in real time (6–9). Many CTC detection methods such as immunomagnetic cell enrichment, PCR-based assays with different selected markers, immunoassays against surface antigens, and membrane filtration by size have evolved in the last decade (4, 10). However, owing to the lack of standardization or reproducibility or the long duration of the assay, most of these techniques are not useful in clinical settings. The CellSearch system, which is the only instrument approved by the US Food and Drug Administration for CTC detection, also has certain shortcomings in clinical application. The expressions of epithelial cell adhesion molecules (EpCAM) and cytokeratins (CK) are highly dynamic in different types or stages of cancer cells, especially in those undergoing EMT, which results in the failure of CTC detection using the CellSearch system (11–13). Moreover, the system can only be used in limited tumors such as breast, colon, and prostatic cancers.

Owing to the rarity of CTCs (almost 1 CTC per million white blood cells), sensitive and specific analytical techniques are needed to enrich and detect them in the peripheral blood (14, 15). In our previous study, we showed that the negative enrichment-fluorescence *in situ* hybridization (NE-FISH) approach has high sensitivity and specificity and enables the rapid isolation and detection of CTCs from whole blood (16–18). Using a cutoff value of 2 CTCs/3.2 ml blood, the sensitivity rates for detecting lung, gastric, and breast cancer were 68.39%, 86.21%, and 76.77%, respectively.

Currently, the clinical prognosis of EC is based on serum tumor marker detection, endoscopy, upper gastrointestinal barium angiography, and computed tomography (CT). As non-invasive methods, the detection of serum tumor markers such as squamous cell carcinoma (SCC) antigen and carcinoembryonic antigen (CEA) is often used to monitor the effectiveness of treatment. Nevertheless, because of their low specificity and accuracy, these markers could not provide an excellent predictive value for patients with EC. Identifying a non-invasive, repeatable, and precise biomarker in the initiation, development, and progression of EC will be helpful in predicting the prognosis of patients with EC and in improving their survival rate. As a liquid biopsy marker for cancer diagnosis, CTCs could also indicate the prognosis in many other cancers such as lung cancer, gastric cancer, and breast cancer (19–22). Most prior studies on the clinical utility of CTCs in EC had been based on small cohorts of subjects. Moreover, few studies had focused on the prediction value of CTCs in the progression and prognosis of EC. In this study, the NE-FISH approach was used to detect CTCs in the peripheral blood of 129 patients with EC. The relationship between CTC numbers or karyotypes and clinical features was also analyzed. Additionally, whether CTC could be used to predict OS and PFS was also investigated.

## Materials and Methods

### Patients and Sample Collection

A total of 129 patients who were newly diagnosed with EC and did not receive any treatment from October 2016 to October 2018 at the Liaocheng People’s Hospital were enrolled in this prospective study. All cancer patients, including 7 with adenocarcinomas and 122 with squamous cell carcinomas, underwent surgery and their disease status was confirmed by histopathological diagnosis. Negative control blood samples were acquired from 17 benign-disease patients and from 75 healthy volunteers who were matched for age and gender to those of the cancer patients (**Table S1**). Patients with benign diseases were diagnosed by histopathology, imaging, and serum tests. Criteria for the healthy group consisted of donors without history of smoking, alcoholism, with family history of cancer, tumors, or other diseases, with normal function of the heart, liver, lung, brain, and with normal results on routine tests for blood, urine, feces, tumor markers, renal and liver function, chest X-ray, and electrocardiography. The experiments conducted in the present study were approved by the Ethics Committee of Liaocheng People’s Hospital (Number: LY2016038), and written informed consent was obtained from each participant included in this study.

Blood samples collected before conducting any treatment were stored at room temperature for CTC analysis within 24 h of collection. To avoid deviation, collection, coding, and detection of all blood samples were performed in a blinded manner by different personnel. Except for 12 patients lost during the follow-up, all EC patients were followed until October 2020 to record the times of progression, recurrence, and death, so as to investigate whether CTCs could be used as an independent prognostic biomarker.

### Enrichment and Identification of CTCs

**Figure 1:**
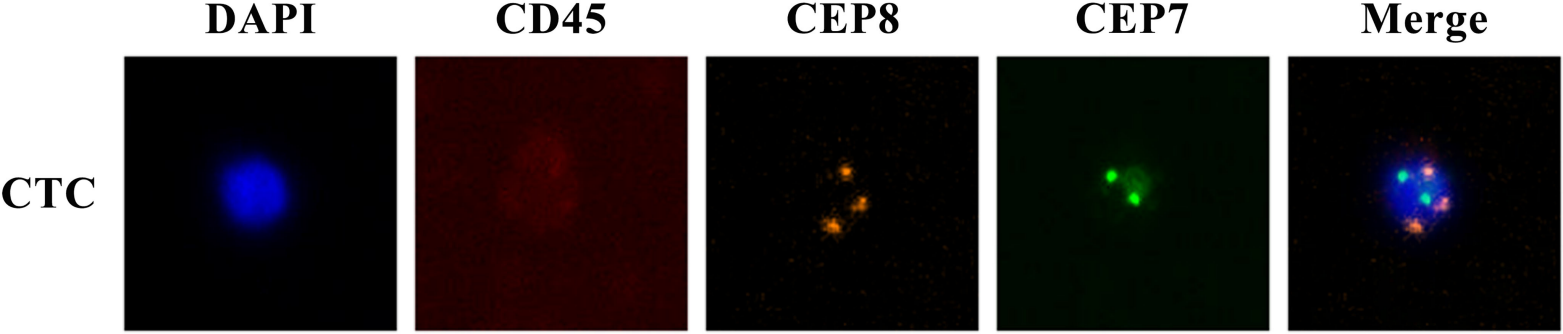
CTC images under fluorescence microscope identified by NE-FISH.

The enrichment and identification of CTCs were performed according to the NE-FISH method, described in our past report (18). Briefly, red blood cells were lysed with CS2 buffer. Then white blood cells were removed by immunomagnetic particles conjugated with anti-leukocyte monoclonal antibodies (anti-CD45) to live rare cells alone. After fixing the cells on the slides, samples were subjected to FISH with a centromere probe (CEP) 8 + 7 (orange + green) and immunostaining with Alexa Fluor 594 conjugated anti-human CD45. Finally, DAPI was used to stain cell nuclei. The cells with DAPI^+^/CD45^-^/chromosome multiploid were identified as CTCs (**Figure 1**). The results were expressed as the number of CTCs per 3.2 ml of whole blood.

### Statistical Analyses

A receiver operating characteristic curve (ROC) was applied to determine the cutoff value for the number of CTCs used to differentiate patients with esophageal cancer from the control subjects. The Mann–Whitney U-test and Kruskal–Wallis H test were applied to evaluate the potential association between CTCs and clinicopathological parameters. Kaplan–Meier survival curves were applied to describe the survival distributions of patients with different levels of CTCs. Cox proportional hazard regression was used to obtain univariate and multivariate hazard ratios for PFS and OS. PFS was defined as the time from blood collection to the time of progression or death. OS was defined as the time from blood collection to death. All statistical analyses were performed using the SPSS version 20.0. All *p* values were examined using two-sided tests, while *p* < 0.05 indicated statistical significance.

## Results

### Patient Characteristics and CTC Count

**Figure 2:**
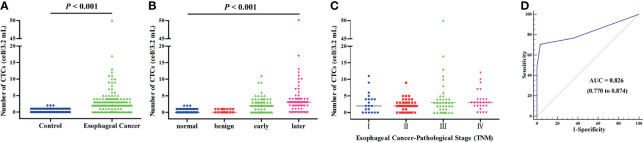
**(A)** The distribution of CTCs in the control and EC groups. **(B)** The distribution of CTCs in healthy, benign, early-stage (I–II), and late-stage (III–IV) patients. **(C)** The distribution of CTCs in different stages of EC patients. **(D)** ROC curve was applied to determine the cutoff value of CTCs in the present study.

The CTC counts in the 129 patients with EC, including seven adenocarcinomas and 122 squamous cell carcinomas, ranged from 0 to 50, with a median value of 2. The number of CTCs detected in the control group ranged from 0 to 2, with a median value of 0, which was significantly lower than that in the patients with EC (*p* < 0.001, **Figure 2A**). A significant difference in the number of CTCs was also noted among healthy, benign, early cancer (I–II), and later cancer (III–IV) donors (*p* < 0.001, **Figure 2B**). In the case of patients with EC, the CTC numbers for the different cancer stages ranged from 0 to 11 for I, 0–9 for II, 0–50 for III, and 0–12 for IV (**Figure 2C**). It is worth noting that the detection of CTCs using the NE-FISH approach could differentiate the patients with EC from benign or healthy controls at a threshold of 2 per 3.2 ml peripheral blood with a sensitivity and specificity of 70.54% and 96.74%, respectively (area under the curve = 0.826, 95% CI 0.770–0.874, *p* < 0.001, **Figure 2D**).

### Association Between CTC Count and Clinicopathological Features

For the patients with EC, the positivity rates based on sex were 69.89% for men (range 0–50, with a median of 2) and 72.22% for women (range 0–12, with a median of 2.5). In 5 patients (71.43%) with esophageal adenocarcinoma and 96 patients (70.49%) with esophageal squamous cell carcinoma, ≥2 CTCs were detected in 3.2 ml of peripheral blood. The positivity rate for patients with distant metastasis was higher than that for those without distant metastasis (80.77% vs. 67.96%, *p* = 0.197). As presented in **Table 1**, the CTC count was associated with tumor depth (T1 vs. T2 vs. T3 vs. T4 *=* 66.67% vs. 37.50% vs. 76.62% vs. 88.89%, *p* = 0.012), but there was no correlation with any other clinicopathological characteristics.

**Table 1:** Relationship of CTC with patient demographics and clinical characteristics.

Characteristics	*n*	Proportion (%)	CTC < 2		CTC ≥ 2		*p*
			n	Proportion (%)	n	Proportion (%)	
Gender							
Male	93	72.09	28	30.11	65	69.89	0.463
Female	36	27.91	10	27.78	26	72.22	
Age							
≥65	79	61.24	21	26.58	58	73.42	0.119
<65	50	38.76	17	34.00	33	66.00	
Histology							
Adenocarcinoma	7	5.43	2	28.57	5	71.43	0.979
Squamous	122	94.57	36	29.51	86	70.49	
Histologic type							
Well differentiated	7	5.43	4	57.12	3	42.86	0.647
Moderately differentiated	68	52.71	22	32.35	46	67.65	
Poorly differentiated	54	41.86	12	22.22	42	77.78	
Vascular invasion							
Absent	57	44.19	17	29.82	40	70.18	0.795
Present	72	55.81	21	29.17	51	70.83	
Tumor location							
Upper	16	12.40	3	18.75	13	81.25	0.218
Middle	58	44.96	19	32.76	39	67.24	
Lower	55	42.64	16	29.09	39	79.91	
Distant metastasis							
M0	103	79.84	33	32.04	70	67.96	0.197
M1	26	20.16	5	19.23	21	80.77	
Tumor depth							
T1	27	20.93	9	33.33	18	66.67	0.012
T2	16	12.40	10	62.50	6	37.50	
T3	77	59.69	18	23.38	59	76.62	
T4	9	6.98	1	11.11	8	88.89	
Lymph node metastasis							
N0	56	43.41	18	32.14	38	67.86	0.095
N1	39	30.23	13	33.33	26	66.67	
N2	24	18.60	6	25.00	18	75.00	
N3	10	7.75	1	10.00	9	90.00	
TNM stage (UIUC)							
I	20	15.50	8	40.00	12	60.00	0.301
II	44	34.11	13	29.55	31	70.45	
III	39	30.23	12	30.77	27	69.23	
IV	26	20.16	5	19.23	21	80.77	

### Association Between CTC Count and Survival (PFS and OS)

**Figure 3:**
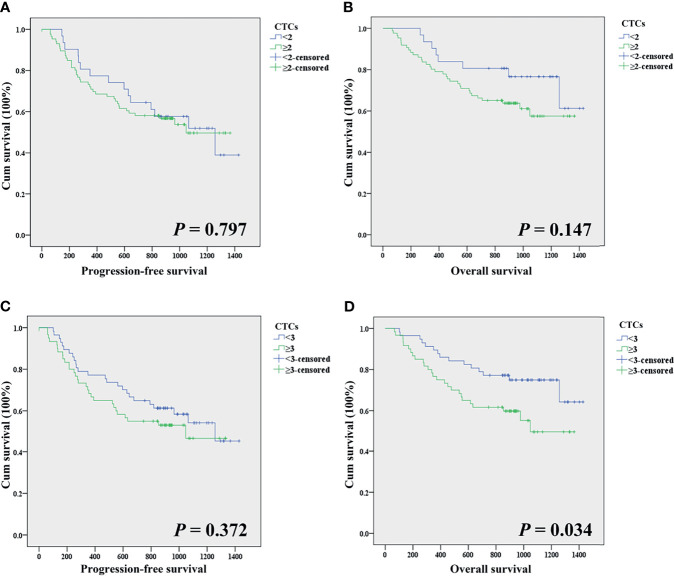
**(A)** Comparison of the progression-free survival time of patients with CTCs <2 and CTCs ≥2. **(B)** Comparison of the overall survival time of patients with CTCs <2 and CTCs ≥2. **(C)** Comparison of the progression-free survival time of patients with CTCs <3 and CTCs ≥3. **(D)** Comparison of the overall survival time of patients with CTCs <3 and CTCs ≥3.

Except for the 12 patients lost during follow-up, 117 patients with EC were followed up in this study. A total of 41 patients (35.04%) died during the follow-up period, and the mortality rates for patients with ≥2 CTCs and <2 CTCs before treatment were 38.37% (33/86) and 25.81% (8/31), respectively. Kaplan–Meier analysis showed that the count of CTCs (≥2) was not associated with either PFS or OS (mean time: 697 days vs. 809 days in **Figure 3A** and 773 days vs. 946 days in **Figure 3B**), while CTCs ≥3 were not linked to PFS (mean time: 668 days vs. 788 days in **Figure 3C**). Interestingly, the OS was significantly shorter for patients with ≥3 CTCs when compared with those having CTCs <3 (mean time: 736 days vs. 906 days, **Figure 3D**, *p* = 0.034).

Univariate analysis, a tool to evaluate which clinical factors could predict the risk for progression or death, showed that sex, vascular invasion, distant metastasis, tumor depth, lymph node metastasis, and TNM stage were the significant prognostic factors for patients with EC (**Table 2**). Multivariate analysis demonstrated that distant metastasis (HR 3.262, 95% CI 1.671–6.369, *p* = 0.001 for PFS; HR 3.759, 95% CI 1.867–7.571, *p* < 0.001 for OS; **Table 2**) was a significant prognostic factor for patients with EC.

**Table 2:** Univariate and multivariate proportional hazard models for OS and PFS.

Factors	PFS				OS			
	Univariate analysis		Multivariate analysis		Univariate analysis		Multivariate analysis	
	HR (95% CI)	*p*-value	HR (95% CI)	*p*-value	HR (95% CI)	*p*-value	HR (95% CI)	*p*-value
Gender: male vs. female	0.463 (0.238~0.899)	0.023	0.498 (0.252~0.984)	0.045	0.419 (0.185~0.945)	0.036	0.516 (0.225~1.183)	0.118
Age: <65 vs. ≥65	0.736 (0.429~1.261)	0.264	0.447 (0.239~0.834)	0.011	0.916 (0.490~1.712)	0.784	0.502 (0.234~1.078)	0.077
Histology: adenocarcinoma vs. squamous	1.450 (0.353~5.960)	0.606	1.103 (0.252~4.818)	0.897	1.197 (0.289~4.966)	0.804	2.792 (0.595~13.104)	0.193
Vascular invasion: absent vs. present	1.826 (1.036~3.220)	0.037	0.900 (0.475~1.704)	0.746	2.020 (1.029~3.962)	0.041	0.796 (0.370~1.711)	0.559
Distant metastasis: M0 vs. M1	4.752 (2.703~8.351)	<0.001	3.262 (1.671~6.369)	0.001	7.946 (4.252~14.848)	<0.001	3.759 (1.867~7.571)	<0.001
Tumor depth: T1+T2 vs. T3+T4	2.513 (1.295~4.877)	0.006	2.235 (0.908~5.504)	0.080	4.752 (1.863~12.118)	0.001	2.260 (0.719~7.102)	0.163
Lymph node metastasis: N0 vs. N1~3	4.746 (2.379~9.468)	<0.001	6.180 (1.511~25.284)	0.011	13.075 (4.025~42.475)	<0.001	4.491 (0.365~55.292)	0.241
TNM stage (UIUC): I+II vs. III+IV	4.036 (2.188~7.444)	<0.001	0.518 (0.136~1.970)	0.335	13.483 (4.786~37.985)	<0.001	2.090 (0.213~20.529)	0.527
CTC count: <2 vs. ≥2	1.082 (0.595~1.967)	0.797	1.048 (0.447~2.459)	0.914	1.761 (0.812~3.821)	0.152	1.373 (0.474~3.973)	0.559
CTC count: <3 vs. ≥3	1.276 (0.746~2.183)	0.373	0.849 (0.396~1.820)	0.674	1.971 (1.042~3.729)	0.037	0.941 (0.383~2.312)	0.894

### Multiploidy Analysis of CTCs

**Figure 4:**
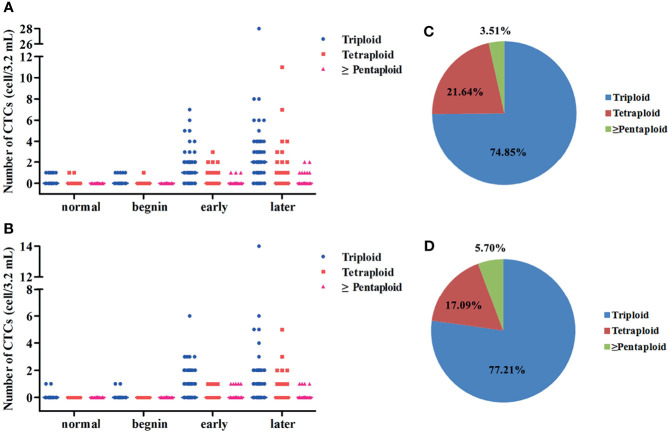
**(A)** Scatter plots for the distribution of CTCs’ chromosome VIII ploidy for normal, benign, early- stage, and late-stage patients. **(B)** Scatter plots for the distribution of CTCs’ chromosome VII ploidy for normal, benign, early-stage, and late-stage patients. **(C)** Pie charts for the proportion of CTC with different types of chromosomal VIII ploidy for EC patients. **(D)** Pie charts for the proportion of CTC with different types of chromosomal VII ploidy for EC patients.

CEP 8 + 7 was used in this study, and one of the criteria for the identification of CTCs was chromosome multiploidy (the fluorescent dots of CEP 8 or CEP 7 under microscope ≥ 3). During the research, we found that the CTCs exhibited varying degrees of chromosomal multiploidy, such as triploidy, tetraploidy, pentaploidy, and beyond in the patients with EC. In contrast, only triploidy and tetraploidy were observed in the control group (**Figures 4A, B** **)**. For normal, benign, early stage, and late stage, the most common aberrations were triploidy for both CEP 8 and CEP 7 (69.23%, 83.33%, 79.17%, and 72.52% for CEP 8; 100%, 100%, 82.54%, and 73.68% for CEP 7). Among the 99 patients with EC in whom chromosome 8 multiploidy was detected in the CTCs, the proportions of different karyotypes were 78.05% for triploidy, 18.39% for tetraploidy, and 3.56% for multiploidy (≥pentaploid) (**Figure 4C**). For the 61 patients in whom chromosome 7 multiploidy was detected in the CTCs, the proportions were 79.98% (triploidy), 13.87% (tetraploidy), and 6.15% (multiploidy), (**Figure 4D**).

The relationship between the proportions of different karyotypes for each patient and the clinicopathological features was also explored in this study. No statistical difference was found between the proportions of CTCs with chromosome 8 ploidy and the clinical characteristics. Patients with distant metastasis had higher proportions of CTCs with chromosome 7 triploidy than those without distant metastasis (*p* = 0.033, **Figure S1A**). Statistical difference was also found between the proportions of CTCs with chromosome 7 triploidy and TNM stage (*p* = 0.002, **Figure S1B**). The proportions of CTCs with chromosome 7 tetraploidy were associated with lymph node metastasis (*p* = 0.041, **Figure S1C**) and TNM stage (*p* = 0.007, **Figure S1D**). Considering the fact that the number of patients in whom chromosome 7 tetraploidy was detected in the CTCs is small, more research involving a larger patient cohort is needed to confirm whether this karyotype holds clinical significance.

## Discussion

Many detection methods such as endoscopy, CT, aer-barium double-contrast radiography, and biopsy are widely used in clinics for the diagnosis of EC (23, 24). However, there continues to be a delay in the diagnosis because of the absence of clinical symptoms in the early stage of EC, which leads to poor prognosis. To improve this situation, several liquid biopsy markers such as CTCs, circulating tumor DNA, microRNA, and long non-coding RNA have been explored in recent years (25, 26). In this research, 129 patients with EC, 17 with benign disease, and 75 healthy volunteers were recruited to investigate whether this liquid biopsy marker could be used as a diagnostic and prognostic tool. When 2 CTCs/3.2 ml of peripheral blood was used as the cutoff value to differentiate the patients with EC from benign or healthy controls, the sensitivity and specificity rates were 70.54% and 96.74%, respectively. For early-stage (I and II) disease, the sensitivity of CTC detection was 67.19%, which is higher than that of most serum tumor markers such as CEA, CA 19-9, and CYFRA 21-1 (27, 28). Furthermore, we found that the CTC counts in different tumor depths exhibited a statistical difference (*p* = 0.012), but this difference could not be found in any other clinicopathological characteristics. In the future, we hope to enroll a larger patient cohort to confirm the association between CTC numbers and clinicopathological parameters.

CT and positron emission tomography, which are the standard imaging methods for monitoring the recurrence sites of solid cancer after treatment, have certain limitations in clinical application owing to their low sensitivity in detecting small lesions. Previous research has demonstrated that CTCs, which have the advantage of non-invasiveness and repeatability, could be used as biomarkers for monitoring the treatment response and prognosis in many types of cancer. Patients with high CTC numbers tend to have a poor survival (12, 29–32). However, to the best of our knowledge, only a few studies have focused on the prognostic value of CTCs in EC, and the levels of CTCs for recurrence, metastasis, or death prediction differed due to variations in the testing methods (33–36). In this study, we followed up 117 patients with EC to record the time from CTC detection to either disease progression or death. Patients with ≤2 CTCs had prolonged OS (mean time: 906 days vs. 736 days, *p* = 0.034), but no significant correlation was found between CTCs and PFS. At the same time, distant metastasis was found to be a significant prognostic factor for patients with EC. These factors are likely to help physicians in focusing on the recursion or progression in patients.

Chromosome aneuploidy, as a hallmark in various tumor cells that drives lethal progression, has been widely used in many CTC detection methods. Several studies have demonstrated that the chromosome ploidy of CTCs holds clinical significance in breast cancer, liver cancer, nasopharyngeal carcinoma, gastric cancer, etc. Chromosome 8 aneuploidy is highly likely to result in recurrence or chemotherapy resistance (37–40). The finding of the present study that tetraploidy is the main aberration in patients before treatment is consistent with the results of previous research (39, 41). Patients in whom a higher proportion of CTCs with chromosome 7 triploidy was detected were more likely to experience distant metastasis. Furthermore, we found that the proportion of CTCs with chromosome 7 triploidy in different TNM stages displayed a statistical difference. Whether triploid CTCs denote progression or treatment resistance in patients with EC needs to be investigated in future research.

The count of CTCs is considered an important index for guiding therapy in EC. Monitoring of CTCs may provide information about the risks of recurrence and metastasis and thus improve the prognosis of EC patients (34–36). The previous studies have shown that CTCs are associated with poor PFS and OS especially when CTC >2 (33, 42, 43). We found that EC patients with CTCs ≥3 had short OS which is consistent with previous studies Moreover, in current research, CTC detection with the NE-FISH approach was achieved with a sensitivity and specificity of 70.54% and 96.74%, respectively. Therefore, the NE-FISH method showed great potential in routine clinical application of patients with EC.

## Conclusion

Overall, the NE-FISH method was used to detect the CTCs in patients with EC in this study. Our results suggest that CTCs detected using this method hold potential clinical application value in the diagnosis of EC. CTC karyotyping demonstrated that the proportion of CTCs with chromosome 7 triploidy makes a significant difference in distant metastasis and TNM stage. Moreover, patients with ≥3 CTCs had a short overall survival and distant metastasis, an independent prognostic factor denoting a poor prognosis.

## Data Availability Statement

The original contributions presented in the study are included in the article/**Supplementary Material**. Further inquiries can be directed to the corresponding authors.

## Ethics Statement

The experiments conducted in the present study were approved by the Ethics Committee of Liaocheng People’s Hospital (Number: LY2016038). The patients/participants provided their written informed consent to participate in this study.

## Author Contributions

YL and ZW analyzed the data and wrote the manuscript. RF and TZ responded to the testing of CTCs. SW performed the follow-up visits and collected data. DY and XT designed the study and revised the manuscript. All authors contributed to manuscript revision and approved the submitted version.

## Funding

This research was funded by the Natural Science Foundation of Shandong Province (Nos. ZR2017MH009 and ZR2021MH215).

## Conflict of Interest

The authors declare that the research was conducted in the absence of any commercial or financial relationships that could be construed as a potential conflict of interest.

## Publisher’s Note

All claims expressed in this article are solely those of the authors and do not necessarily represent those of their affiliated organizations, or those of the publisher, the editors and the reviewers. Any product that may be evaluated in this article, or claim that may be made by its manufacturer, is not guaranteed or endorsed by the publisher.
