# Supplementary material for: Clinical Utility of Circulating Tumor Cells in Patients With Esophageal Cancer
Source: Front Oncol. 2022 Mar 21;12:828368. doi: 10.3389/fonc.2022.828368 (PMC8977550; doi:10.3389/fonc.2022.828368)
Supplement: Supplementary file 2 [file DataSheet_2.docx]

Clinical utility of circulating tumor cells in patients with esophageal cancer

*Yang Li^1^* ^†^*, Zhenxing Wang^2^* ^†^*, Rao Fu^1^, Shuang Wang^1^, Tingting Zhang^1^, Xudong Tian^2*^, Dawei Yang^1*^*

*^1^ Zhong Yuan Academy of Biological Medicine, Liaocheng People's Hospital, Liaocheng, Shandong, P. R. China*

*^2^ Department of Thoracic Surgery, Liaocheng People's Hospital, Liaocheng, Shandong, P. R. China*

^†^ *These authors contributed equally to this work.*

**** Correspondence:****Dr. Dawei Yang (Primary)*[*yangdawei775@163.com*](mailto:yangdawei775@163.com)

*Dr. Xudong Tian (Secondary)*

*40756518@qq.com*

**TABLE S1**︱Comparison of basic characteristics between control and cancer group.

|  | **Esophageal cancer** | | ***P*** |
| --- | --- | --- | --- |
|  | **Control** | **Test** |  |
| Gender |  |  |  |
| Male (%) | 69.57 | 72.09 | 0.683 |
| Female (%) | 30.43 | 27.91 |  |
| Age (y), mean ± SD | 64.00±11.29 | 65.13±9.16 |  |
| ≥65 (%) | 44.57 | 38.76 | 0.091 |
| <65 (%) | 55.43 | 61.24 |  |


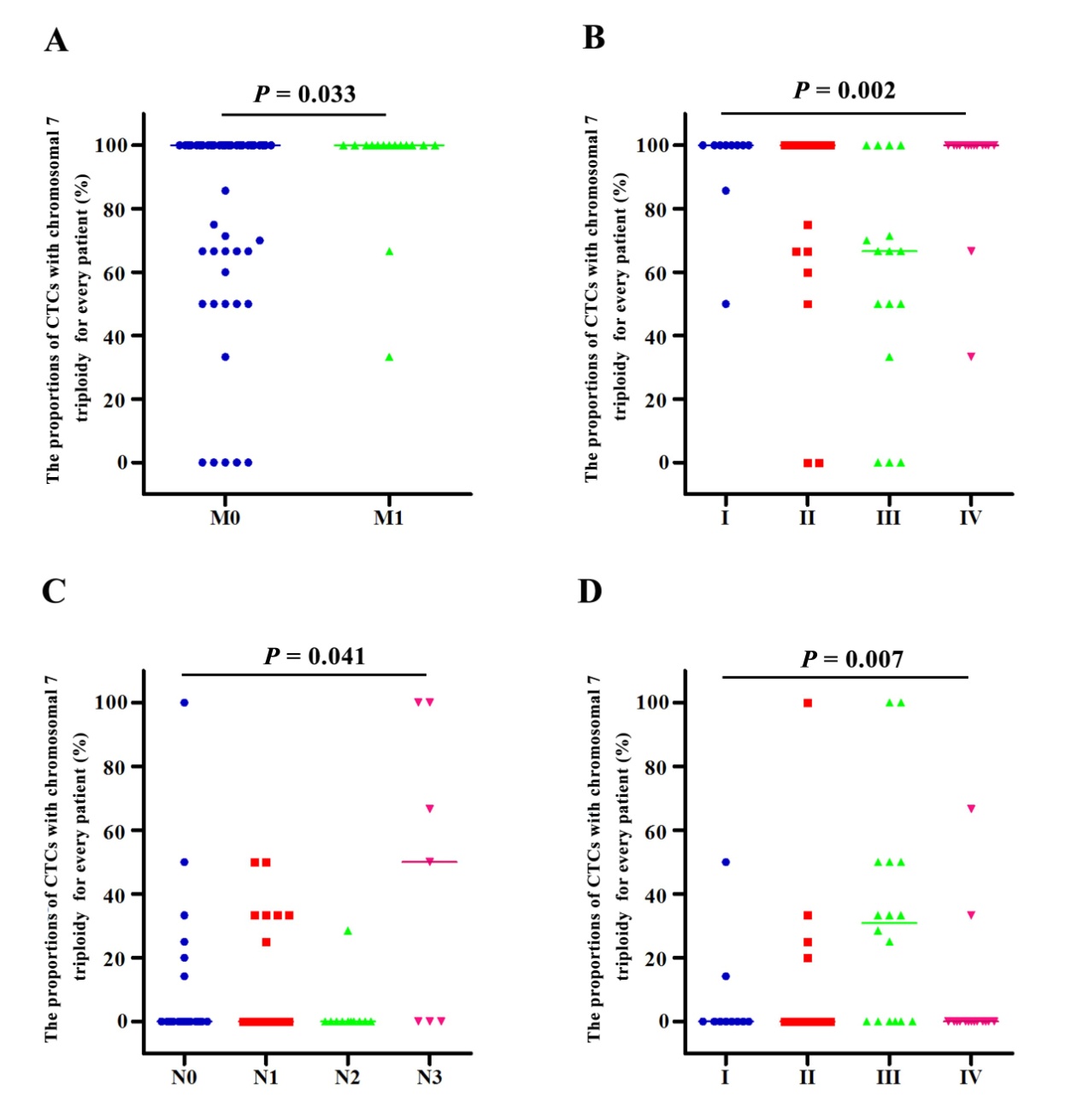


**Supplementary Figure 1.** **(A)** Scatter plots for the proportions of CTCs with chromosomal 7 triploidy for patients with or without distant metastasis. **(B)** Scatter plots for the proportions of CTCs with chromosomal 7 triploidy for patients with different TNM stage. **(C)** Scatter plots for the proportions of CTCs with chromosomal 7 tetraploidy for patients with different lymph node metastasis. **(D)** Scatter plots for the proportions of CTCs with chromosomal 7 tetraploidy for patients with different TNM stage.
